# Design and Evaluation of a Polymer Support Fluid in a Soil–Rock Mixture

**DOI:** 10.3390/polym14071402

**Published:** 2022-03-30

**Authors:** Chunye Ying, Xinli Hu, Peng Xia, Haiyan Zhang

**Affiliations:** Faculty of Engineering, China University of Geosciences, Wuhan 430074, China; cy.ying@cug.edu.cn (C.Y.); zhanghaiyan@cug.edu.cn (H.Z.)

**Keywords:** polymer, soil–rock mixture, bored piles, viscosity

## Abstract

Soil–rock mixtures are commonly encountered in the construction of bored piles. Conventional bentonite support fluids have disadvantages, such as more significant environmental impacts, more complex mixing, bigger site footprint, weaker foundation performance, and overall low economies. The present study conducted a comprehensive investigation of partially hydrolyzed polyacrylamide (PHPA) polymer fluids, an alternative to bentonite ones, to drill into a soil-limestone mixture. The fluid flow pattern, aging behavior, and the influence of finer silty clay on polymer fluid were explored. The test results showed that polymer fluids were reasonably well fitted to the power-law model and were a good alternative to the conventional bentonite ones. In terms of their aging behavior, the remaining active viscosity of the polymer was at least 70% after a prolonged aging time of up to 30 days, showing the effective on-site use of polymer fluids. The mixing of silty clay significantly reduced the apparent viscosity of polymer fluids, with 10% silty clay causing a viscosity reduction of 76%, indicating the importance of fluid control in drilling these materials. A polymer formula, water + 0.08%PHPA + 0.1~0.5%Na_2_CO_3_, was proposed and was verified by drilling into a soil–limestone mixture. The polymer fluids led to small radial displacements around the boreholes with a high drilling quality. This work would be helpful for consultants and contractors designing and constructing bored piles in soil and rock mixtures utilizing polymer fluids.

## 1. Introduction

In the oil and gas industry, soil and rock mixtures, which are an essential material composition of overburdens, are often treated as a “black box” and are quickly drilled through to get to the reservoir [1]. As a result, much attention has been paid to the deeper reservoir than the shallower soil and rock mixtures in petroleum science. However, these materials are commonly encountered in the works of geotechnical construction [2], where the bored piles and support fluids to drill these materials are usually the focus of attention in this case.

Bentonite support fluids have been utilized for more than 60 years in construction projects worldwide since the pioneering work of Veder [3]. However, their long-term usage also raised several problems, such as more significant environmental impact, more complex mixing, a larger site footprint, weaker foundation performance, and overall low economies [4]. With material and technology development, synthetic polymer fluids notably overcome the above-mentioned disadvantages of the bentonite ones [5]. As a result, polymer fluids are increasingly being used as a complete replacement for the conventional bentonite slurries in civil engineering. Aqueous solutions of partially hydrolyzed polyacrylamides (PHPA) are among the most widely utilized products [6].

The polymer’s performance as a borehole stabilizer under different ground conditions has been widely studied. The piles in chalk in Norwich, Norfolk, constructed with polymer fluid, behaved better in shaft resistance than those drilled with water and bentonite fluid [7], possibly due to the great supportability of polymer fluid to the pile bore. Wheeler [8] utilized polymer fluids in stiff clay and dense sand in London. It was found that the piles constructed using polymer fluids did not reduce pile shaft resistance, and increasing the construction time from 12 h to 37 h showed a negligible effect on pile performance. These findings were verified by Lam et al. [9] and Lam [10], who observed that piles formed with polymer fluid significantly outperformed piles supported by bentonite fluid, and little difference was found between piles drilled within 7.5 h and 26 h. Bustamante et al. [11] investigated piles constructed with polymer fluid in pyroclastic soil consisting of pumice and lapilli, and they concluded that polymer fluid showed no adverse effect on pile shaft resistance in the pyroclastic soils. Similar successful cases of polymer fluids as a pile bore stabilizer can also be found in sand [12,13].

However, few studies have been carried out utilizing polymer support fluids to drill soil and rock mixtures. This situation is possibly attributed to the common perception that only bentonite support fluid can stabilize gravelly soil due to its inherent ability to form a layer of filter cake to seal the soil surface. Lesemann [14] reported the successful use of three types of polymer fluids, i.e., polyacrylamide (PAA), carboxymethyl cellulose (CAM), and xanthan gum (XAM), in a sand-gravel mixture in Munich, Germany, which disproved the above-mentioned common perception. Their findings also indicated that sufficient viscosity coupled with the multi-point adsorption on the borehole wall as well as clogging the soil pores with the finer excavated materials [15] was the key to the successful application of polymers in the sand–gravel mixture. To date, the suitability evaluation of the most widely utilized PHPA in drilling soil and rock mixtures is still poorly studied in civil engineering, which restricts the application of polymers. Therefore, it is of great significance that we explore the possible application of PHPA fluids to drill commonly encountered soil and rock mixtures in civil engineering.

The following three aspects are the most important for polymer support fluid evaluation in soil and rock mixtures. First, the proper mathematical models are used to interpret the flow behavior of polymer support fluids [16,17]. Second, the polymer fluid aging behavior is critical as these fluids may be used for weeks to months. Nevertheless, no consistent conclusions can be applied in civil engineering; some have reported an initial rapid decline in viscosity [18], some declared a significant rise in viscosity [19], and some found a slow decrease [20], while others found a first slight decrease and a further reduction to constant in viscosity [15]. Lastly, during the excavation work in soil and rock mixtures, the finer soil particles will inevitably fall into the borehole, which causes soil absorption on polymer chains, resulting in a reduction in the active polymer in a support fluid with use [21]. Therefore, it is crucial to evaluate the influence of silty clay on polymer fluid when drilling soil and rock mixtures.

In the present study, screening tests were performed first to select a proper dosage of polymer fluids by producing the same Marsh funnel viscosity with the conventional bentonite slurries. Thereafter, the polymer fluid flow pattern, aging behavior, and effect of polymer fluid mixed with silty clay were investigated. Finally, the proposed polymer fluid formula and water were used to form boreholes in a soil–limestone mixture. The horizontal radial displacements around these boreholes and the formed grouted piles were analyzed to verify the polymer fluid formula. This research work can be used as a reference for bored piles designers and contractors to drill soil and rock mixtures using polymer fluids.

## 2. Materials and Methods

### 2.1. Materials

#### 2.1.1. Polymer

**Figure 1:**
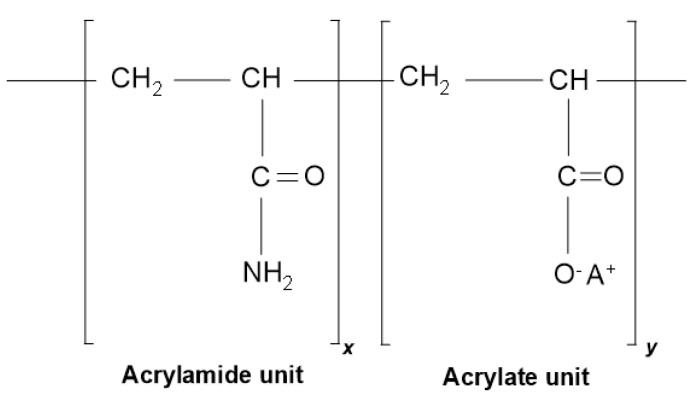
Chemical structure of PHPA.

The polymer used in this paper was white granular powder, which is an acrylamide/sodium acrylate copolymer (partially hydrolyzed polyacrylamide, PHPA). PHPA comprises repeating units of acrylamides and acrylates. The chemical structure of PHPA is shown in Figure 1, where A is a cation, typically sodium, and the values of x and y depend on the product. It was purchased from Tianjin Sami Chemical Co., Ltd., Tianjin, China. The molecular weight and the degree of hydrolysis were 20 × 10^6^ g/mol and 40%, respectively. As a result of its high molecular weight and high charge, this kind of polymer was chosen as a representative of many other PHPAs used in practice. According to the supplier and project site experience, the recommended dosage of PHPA should be between 0.02% and 0.07% during the construction of clay, while in coarse sand, small gravel, and cobbles, the dosage can be increased to 0.2% or even higher according to the actual situation.

#### 2.1.2. Bentonite

The bentonite used was sodium-activated bentonite, which was supplied by Anji Yiguo Co., Ltd., Huzhou, China, with a particle size of 325 mesh and a cation exchange capacity of 89 meq/100 g. The XRD analyses of the bentonite indicated that the content of montmorillonite was 82%, illite was 6%, quartz was 3%, feldspar was 4%, and calcite was 5%.

#### 2.1.3. Soil–Limestone Mixture

A human-made soil–limestone mixture developed by Ying et al. [22] was utilized. The physical properties of the mixture are summarized in Table 1. The fine particles were silty clay, and the gravelly component of the mixture was fresh (nonweathered) limestone fragments. A detailed description and the mineral composition of the mixture can be seen in their work [22].

#### 2.1.4. Silty Clay

Silty clay was a fine particle in the soil–limestone mixture. It was chosen as loose soil particles around the borehole to evaluate the impact of soil particles falling into the polymer fluid. The physical properties of the silty clay are shown in Table 2.

#### 2.1.5. Solvent

As PHPA is an anionic polymer, it is susceptible to the ionic strength of the solvent, so polymer fluid is highly affected by the composition of mixed water. Therefore, in this paper, deionized water was used to prepare the test solutions to ensure the repeatability of the test results.

### 2.2. Methods

#### 2.2.1. Preparation of Polymer Fluid

In order to avoid the effect of high-speed stirring on polymer solution, the method recommended by Lam et al. [6] was adopted to prepare polymer fluids. The method was as follows: First, a vortex in the mixed water was created at a speed of 500 r/min, and then the required amount of PHPA was slowly sifted into the vortex; second, after the completion of the addition, to minimize unnecessary shear to the fluid, the speed was reduced to 200 r/min; lastly, the solution was stirred for approximately 45 min. The slurry was left to stand overnight before use to ensure the consistency of the polymer fluid properties before the test.

#### 2.2.2. Preparation of Bentonite Fluid

A high-speed mixer was used to stir at a speed of 2000 r/min to ensure that the bentonite was thoroughly hydrated. The required amount of bentonite was slowly added to the cup. After the mixing was complete, the bentonite fluid was kept at room temperature overnight to ensure full hydration.

#### 2.2.3. Preparation of Polymer Mixed Silty Clay

First, polymer fluid was prepared according to the preparation method of polymer fluid. Then, referring to the preparation method of bentonite fluid, silty clay fluid was designed. After the two slurries were hydrated overnight, respectively, the silty clay fluid and the polymer fluid were mixed at a volume ratio of 1:1 to form a series of mixtures. The polymer fluid concentration was 0.08% in the mix, and the silty clay concentration ranged from zero to 10% to simulate the mixing of different addition amounts of silty clay in the polymer fluid. The polymer–silty clay mixture was left to stand overnight to ensure uniform mixing.

#### 2.2.4. Observation of Polymer Microstructure

A certain amount of polymer fluid with a concentration of 0.08% was prepared according to the above preparation method. To reveal the polymer microstructure, the sample preparation method employed by Zhu et al. [23] was adopted in this paper, which is briefly described as follows: (i) a small droplet (approximately 2 μL) of the polymer solution was pre-frozen on liquid nitrogen for 30 s, and then the sample was immersed in liquid nitrogen for 2~3 min; (ii) after freezing in liquid nitrogen, the sample was quickly transferred to a vacuum freeze dryer at −30 °C, vacuumed and dried for 48 h, and removed for gold-sprayed coating; (iii) the droplet of the prepared fluid was examined using a scanning electron microscope (SEM).

#### 2.2.5. Fluid Performance Test

The samples were tested for their density, Marsh funnel time, and apparent viscosity over the shear rate range of 5~1022 s^−1^. The fluid density was measured with an NB-1 mud hydrometer, the Marsh funnel time was qualified with a Marsh funnel viscometer, and the apparent viscosity was tested with a ZNN-D6 six-speed rotary viscometer.

## 3. Results

### 3.1. Screening Tests on Polymer Concentration

**Figure 2:**
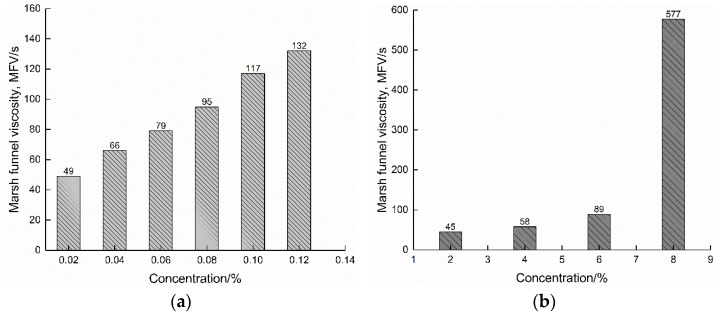
Marsh funnel viscosity of two fluids: (**a**) polymer fluid; (**b**) bentonite fluid.

Figure 2 shows the Marsh funnel times of different concentrations of polymer and bentonite fluids. An amount of 0.02~0.12% added polymer was selected, and the concentration of bentonite was 2~10%. In general, with the increase in the dosage of the two fluids, their Marsh funnel viscosities increased. The effect of concentration on PHPA polymer fluid viscosity can be explained as follows: a higher concentration gives more chain entanglements, thus increasing the strength of the PHPA molecular network [16]. As the polymer concentration increases, the number of polymer chains increases, and the entanglements resist flow increases, manifested as an increase in viscosity [4,6]. As the amount of bentonite increased from 2% to 10%, the viscosity of the bentonite fluid continued to increase, which was caused by the increase in the number of bentonite particles in the colloidal suspension system formed by the clay–water system [24]. It should be noted that when the dosage of bentonite was 8%, the viscosity was 577 s, while when the dosage of bentonite was 10%, the fluid blocked the nozzle at the lower part of the funnel, so the Marsh funnel time could not be measured in this situation.

It could be seen that when the polymer concentration was 0.08%, and the bentonite concentration was 6%, the viscosity values of the two fluids were close, which were 95 s and 89 s, respectively. In other words, only in Marsh funnel viscosity, the 0.08% polymer and 6% bentonite fluids behaved similarly. This means that the amount of bentonite is approximately 75 times that of the polymer, so in terms of material purchase, the polymer can be purchased at one time, while bentonite often needs to be bought in batches many times due to its large demand and limited construction site. However, it should be noted that Marsh funnel viscosity is only a single-point result and one of the parameters representing the comprehensive fluidity of fluid. Therefore, it is not suitable for the development of a detailed understanding of fluid behavior under continuously changing conditions in the field. In light of this, the shear stress and apparent viscosity of bentonite and polymer fluids at different shear rates were measured using a ZNN-D6 six-speed rotary viscometer.

### 3.2. Fluids Flow Patterns

**Figure 3:**
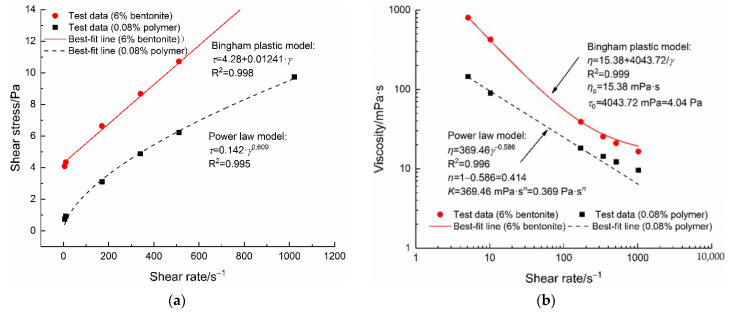
Rheological properties of 6% bentonite and 0.08% polymer fluids: (**a**) shear stress-shear rate relationship; (**b**) apparent viscosity-shear rate relationship.

As illustrated in Figure 3a, the shear stress versus the shear rate plot of 6% bentonite and 0.08% polymer differed. For the former, it was reasonably well fitted to the Bingham plastic model, while for the polymer fluid, the power-law model was suitable. Figure 3b showed the apparent viscosity versus shear rate. Again, it can be seen that the Bingham plastic and the power-law models were well fitted to bentonite and polymer fluids, respectively.

For both bentonite and polymer fluids, within the shear rate range from 5.11 s^−1^ to 1022 s^−1^, the greater the shear rate, the greater the shear stress (Figure 3a). Under the condition of low shear rate (5.11 s^−^^1^), the shear stress promoting the flow of a 6% bentonite fluid was 4.04 Pa, which was greater than that of a 0.08% polymer fluid, which needed 0.74 Pa to promote the fluid. For the bentonite fluid, the swelling properties and the interparticle attractive energy can form a three-dimensional strong yet deformable structure [25,26]. A polymer fluid possesses a three-dimensional molecular network structure with intertwined and entangled chains [16]. In a bentonite fluid, particle interaction forces such as the van der Waals forces are responsible for the formation of flocs and aggregates, which can withstand the flow. In a polymer fluid, the entanglements of the long-chain molecules resist flow. When sheared at 5.11 s^−1^, a higher shear stress of 4.04 Pa was needed for the bentonite fluid to break up its structure. This phenomenon was probably due to the fact that, compared with the 0.08% PHPA polymer fluid, the inner structure of the 6% bentonite fluid can be more robust.

Figure 3b showed a shear-thinning behavior for the polymer fluid, which was typical for polymer fluids prepared at other concentrations within the same shear rate range. Bentonite fluid had a higher apparent viscosity than polymer fluid, especially at low shear rates. For example, when the shear rate was 5.11 s^−1^, the apparent viscosity of bentonite fluid was 800 mPa·s, and that of polymer fluid was 145 mPa·s. The difference gradually decreased as the shear rate increased.

### 3.3. Aging Behavior of Polymer Fluids

**Figure 4:**
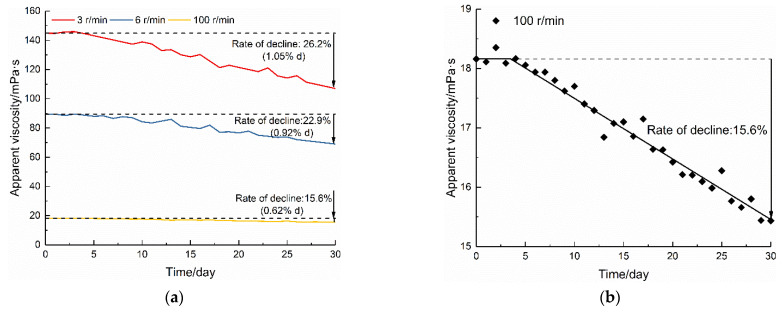
Viscosity-time profiles for polymer fluids: (**a**) different rotor speeds; (**b**) at 100 r/min.

The on-site polymer drilling fluid may be used for a long time after preparation, so 0.08% polymer drilling fluid was prepared and kept for 0~30 days to evaluate the effect of aging on the apparent viscosity of PHPA. The above-mentioned ZNN-D6 six-speed rotary viscometer was selected to test the apparent viscosity of polymer fluids after aging for different days. The results obtained at different viscometer measurement speeds (3 r/min, 6 r/min, and 100 r/min, corresponding to the shear rates of 5.11 s^−1^, 10.22 s^−1^, and 170.3 s^−1^, respectively) were used for analysis, as shown in Figure 4.

In general, the apparent viscosity was very different at the three speeds. This phenomenon is to be expected and can be explained by the shear-thinning non-Newtonian behavior of polymer fluid, so that the higher the shear rate, the lower the apparent viscosity. Due to the large difference between the apparent viscosity values at each speed (Figure 4a), the test data of 100 r/min are plotted in Figure 4b. In terms of its aging behavior, the apparent viscosity of the polymer fluid remained unchanged during the first four to five days and then decreased with the increase in aging time. After the same aging for 30 days, the apparent viscosity of the polymer fluid decreased by 26.2% (1.05%/d) at three r/min, by 22.9% (0.92%/d) at six r/min, and by 15.6% (0.62%/d) at 100 r/min. These results showed that a higher spindle speed did not lead to extra shear degradation, consistent with the finding reported by Lam and Jefferis [27]. The gradual reduction in the apparent viscosity is presumably due to chain disentanglement caused by a conformational change in the polymer molecules [28].

The aging behavior presented in our study is in line with the research carried out by Lam and Jefferis [6] although differs from that of [18,29], who described an initial rapid reduction in viscosity. The discrepancy between our results and theirs is discussed later.

### 3.4. Effect of Polymer Fluids Mixing with Silty Clay

**Figure 5:**
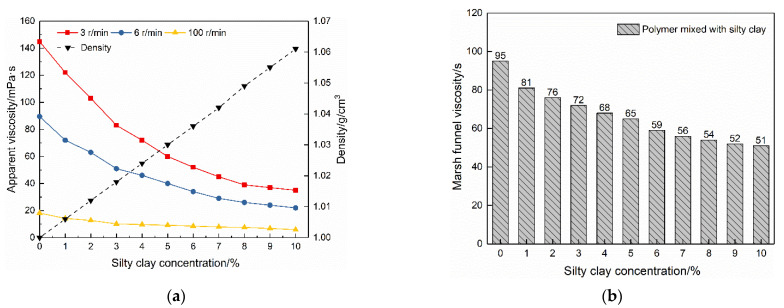
Effect of adding silty clay on polymer fluids: (**a**) changes in apparent viscosity and density; (**b**) changes in Marsh funnel viscosity.

The polymer–silty clay mixture was prepared using the method described above to evaluate the effect of polymer fluid mixed with silty clay. It was left to stand overnight, and the upper solid-free supernatant was taken to test its apparent viscosity. The density of the mixture was calculated with the constant density of the pure polymer fluid of 1.0 g/cm^3^. The effect of adding silty clay on the polymer fluid is shown in Figure 5.

As the concentration of silty clay increased, the apparent viscosity of the mixture showed a downward trend, while the density gradually increased. For example, when the rotation speed was three r/min, and the silty clay addition amount was only 3% (30 kg/m^3^), the apparent viscosity of the polymer fluid dropped from 144.9 mPa·s to 72 mPa·s, a decrease of approximately 50.3%. This phenomenon means the loss of more than 50% of the active ingredients in the polymer fluid. When adding 3% silty clay, the specific gravity of the mixture was increased from 1.0 g/cm^3^ to 1.018 g/cm^3^. At first sight, the apparent viscosity of the polymer fluid decreased significantly at a low speed. In fact, at the end of this test, that is, when the dosage of silty clay was 10%, the decrease in apparent viscosity at each speed was close. Specifically, the apparent viscosity reduction rate was 75.85% at 3 r/min, 75.42% at 6 r/min, and 67.51% at 100 r/min. These results indicated that a higher spindle speed did not lead to extra shear degradation. The gradual reduction in the apparent viscosity caused by silty clay was associated with its sorption on the polymer molecular chains. The sorption reduced the active concentration of the polymer, manifesting as the apparent viscosity reduction [21].

The solid-free supernatants were also tested with the Marsh funnel. The results are shown in Figure 5b. The Marsh funnel viscosity decreased with the increase in silty clay dosage, consistent with the results achieved from a six-speed rotary viscometer. However, in terms of the rate of viscosity reduction, the results found by the Marsh funnel were lower than those of the rotary viscometer. For example, when the dosage of silty clay was 10%, the decrease in apparent viscosity achieved from the Marsh funnel was 46.3%, lower than 75.85% at three r/min, 75.42% at six r/min, and 67.51% at 100 r/min obtained using the rotary viscometer. These results suggested that the Marsh funnel can be used as a viscosity management tool on site, however, its test results may differ from the actual situation.

### 3.5. Polymer Fluid Formula

As analyzed before, water + 0.08% PHPA performed almost the same with water + 6% bentonite in terms of the Marsh funnel viscosity. Section 3.2, Section 3.3 and Section 3.4 tested the polymer fluid flow pattern, aging behavior, and the impact of polymer fluid mixing with silty clay. These results would play a guiding role in the use of the polymer on site.

Since PHPA drilling fluid is always used in an alkaline environment, it is necessary to test the pH value of the fluid in a timely manner. In an actual job site, Na_2_CO_3_ is added into the mixed water first, and then the pH value of the solution is adjusted to approximately 10. In this way, the dual purpose of removing the hardness of water (mainly caused by Ca^2+^ and Mg^2+^ in water) and creating an alkaline environment for the use of polymer fluids can be achieved. Zhang et al. [30] pointed out that 0.3~0.5% Na_2_CO_3_ can increase the pH in the polymer fluids to above 10. Lam and Jefferis [31] observed that 0.09% Na_2_CO_3_ is sufficient to increase the pH to 10 and remove approximately 90% of the water hardness. In this paper, 0.1~0.5% is the recommended dosage of Na_2_CO_3_. In practical engineering, the simple dosage screening of Na_2_CO_3_ can be carried out according to water quality, tested with pH test paper or a pH meter to select a reasonable value.

Within a certain range of drilling depth, low-density PHPA drilling fluid can be used on the premise of ensuring the safety of tripping the drilling tool and placing the reinforcement cage. In this regard, a polymer formula, water + 0.08%PHPA + 0.1~0.5%Na_2_CO_3_ was proposed.

## 4. Discussion

In this paper, we present a comprehensive investigation of polymer fluids to drill into a soil–limestone mixture. The results showed that the apparent viscosity remained almost constant during the first four to five days and then decreased with aging time. The inconsistency between our results and others [15,18,19,20] may be due to the differences in sample preparation procedures, test methods, and material properties. After a prolonged aging time of up to 30 days, the remaining active viscosity of the polymer was at least 70%, consistent with the study carried out by Lam and Jefferis [27], who reported that 75% of the original viscosity remained after aging. This finding will promote the confidence of on-site construction workers to use polymer fluids.

In addition, the test results showed that, as the dosage of silty clay increased from 3% to 10%, the rate of viscosity reduction was from 50% to almost 76%. Lam and Jefferis [21] investigated a 0.08% PHPA polymer fluid, and they found that over 80% of the active polymer was lost when the dosage of London clay was around 15%. Shrivastava et al. [4] found that when the silt content was 5%, the rate of viscosity reduction was approximately 15% for a 0.1% polymer fluid. The difference in material properties and clay content plays an essential role in affecting our results and theirs. One finding is that the effect of adding silty clay on the polymer fluid was a reduction in the apparent viscosity, which can be efficiently eliminated by strictly controlling the content of silty clay and replenishing new slurry in time [27].

The two main findings confirm that polymer fluids can be used in a soil–limestone mixture. However, the mechanism of the polymer fluid as a borehole stabilizer and the practical use performance of the polymer fluid formula need to be further explained and verified.

### 4.1. Microstructure of the Polymer Fluid

**Figure 6:**
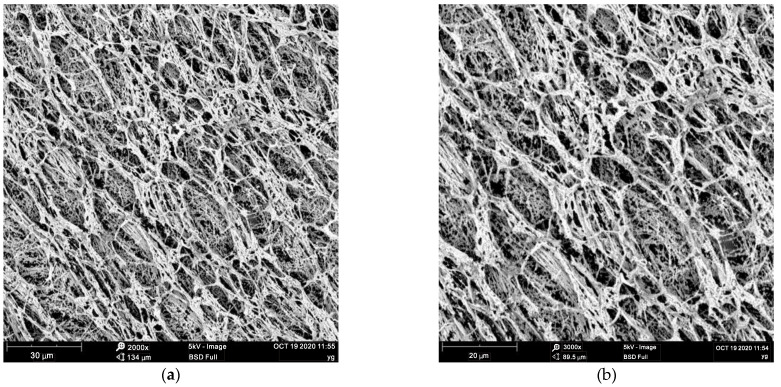
Electron micrograph of a polymer fluid: (**a**) 2000 times magnification; (**b**) 3000 times magnification.

Since the rheological property of a polymer fluid is highly dependent on the microstructure of the fluid, scanning electron microscopy (SEM) was used to detect the prepared fluid droplets. According to the above preparation method, 0.08% polymer fluid was prepared. The microstructures of the polymer fluid magnified 2000 times and 3000 times are shown in Figure 6a,b, respectively. It can be seen that the polymer possesses a three-dimensional molecular network structure with intertwined and entangled chains instead of individual and independent chains.

The micrograph also shows that although the polymer molecules are entangled with each other, the structure appears to be rather stiff and does not curl up. This microscopic feature is significant as if the molecular chains are curled, the interaction between the molecular chains will be reduced, affecting the fluid properties. Due to the electrostatic repulsion between anionic charges distributed along the polymer chains, the curling between polymer molecules is inhibited [16]. The mechanism of the polymer fluid as a borehole stabilizer from its fluid microstructure is described later.

### 4.2. Validation of the Polymer Fluid Formula

**Figure 7:**
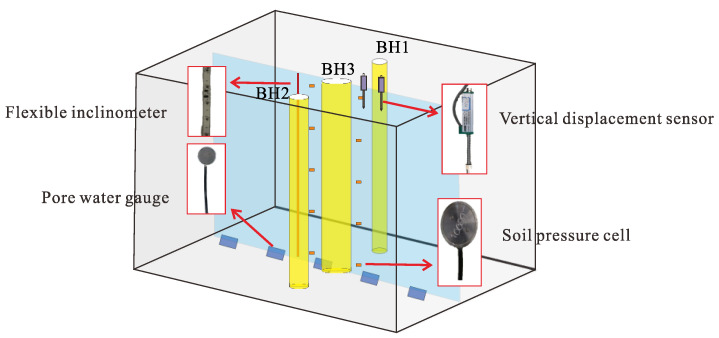
Three-dimensional schematic diagram of instrument layout around BH3.

A model test platform, developed by Ying et al. [22], configured with multiple sensors (Figure 7), i.e., vertical displacement sensors, flexible inclinometers, pore water gauges, and soil pressure cells, was used to verify the polymer fluid formula to drill into a soil–limestone mixture. A detailed description of the platform can be seen in their work [22]. The above-mentioned human-made soil–limestone mixture was used to construct the model. The polymer formula, water + 0.08%PHPA + 0.1~0.5%Na_2_CO_3_, was verified. In this test, the dosage of Na_2_CO_3_ was 0.3%.

**Figure 8:**
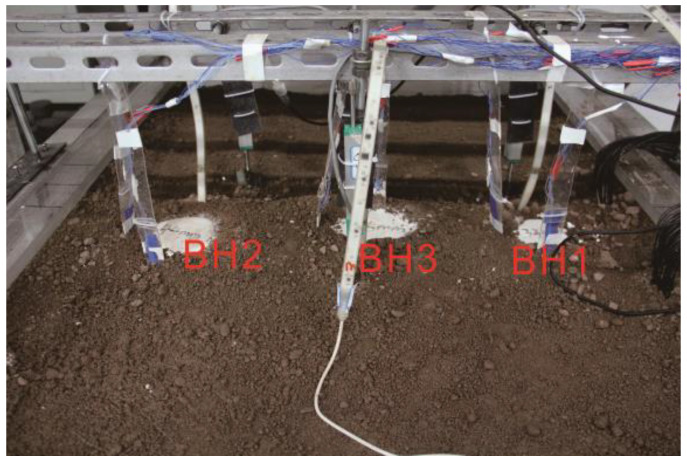
Poured holes after drilling.

As shown in Figure 8, boreholes BH1, BH2, and BH3 had the same depth of 900 mm, but different diameters of 32 mm, 44 mm, and 56 mm, respectively. The distances of BH1 and BH3 centers differed by 4 *d* (*d* equals 56 mm); the same was true for BH2 and BH3 (Figure 8). The flexible inclinometer probe was placed 50 mm in front of the BH3 hole wall parallel to the borehole axis. The flexible inclinometer probe arrangement was the same for BH2.

Herein, boreholes BH2 and BH3 were drilled using water and the polymer fluid formula, respectively. The radial displacement during drilling and piles formed in BH2 and BH3 was investigated to evaluate the performance of the fluids.

#### 4.2.1. Horizontal Radial Displacements

**Figure 9:**
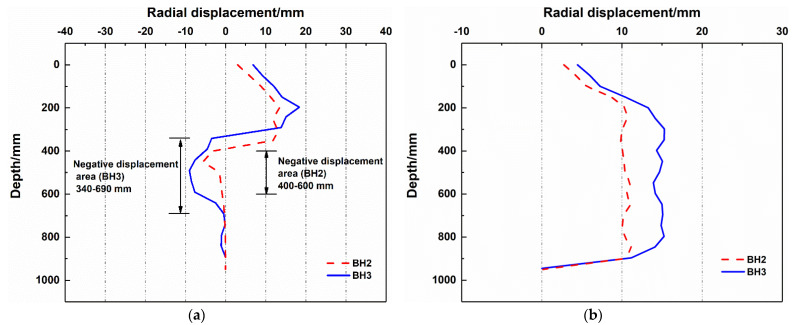
Radial displacement around boreholes with different drilling fluids: (**a**) water; (**b**) polymer fluids.

Figure 9 plots the radial displacement around boreholes BH2 and BH3. Clean water and polymer fluid were used to drill them, respectively.

When water was used to form the boreholes, noticeable negative displacement areas were found to appear in the lower part of the borehole (Figure 9a). As previously analyzed by Ying et al. [22], the negative displacement area indicated movement towards the borehole. In contrast, only positive displacement induced by drilling was observed as polymer fluid was used (Figure 9b). In terms of the positive radial displacement, the use of polymer fluid reduced the radial displacement around the borehole compared with water. For example, when drilling BH3 with clean water, the maximum radial displacement around the hole was 18.69 mm. When drilling with polymer fluid, the maximum radial displacement was 15.29 mm, which was closely related to the excellent lubrication of the polymer fluid [32]. The friction during drilling with polymer drilling fluid was relatively lower than that with clean water so that the soil deformation around the hole caused by drilling tools was small.

#### 4.2.2. Comparison of Pile Morphology after Drilling

**Figure 10:**
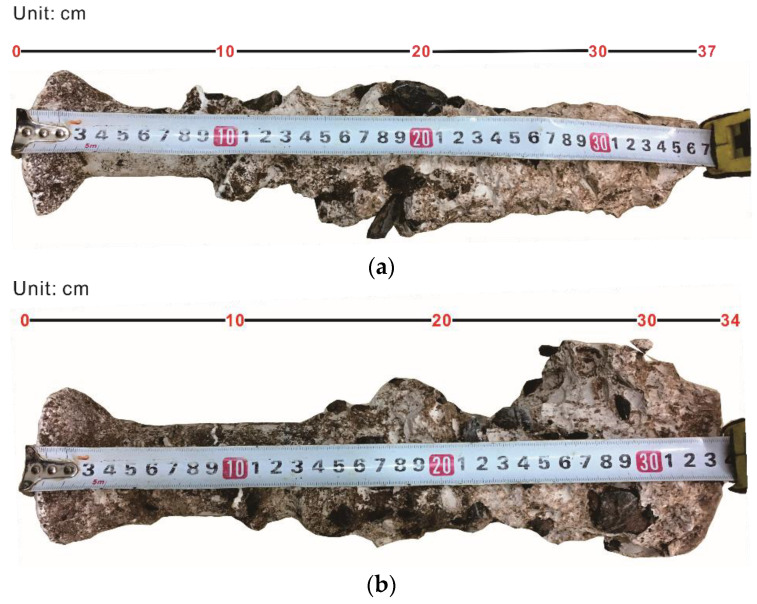

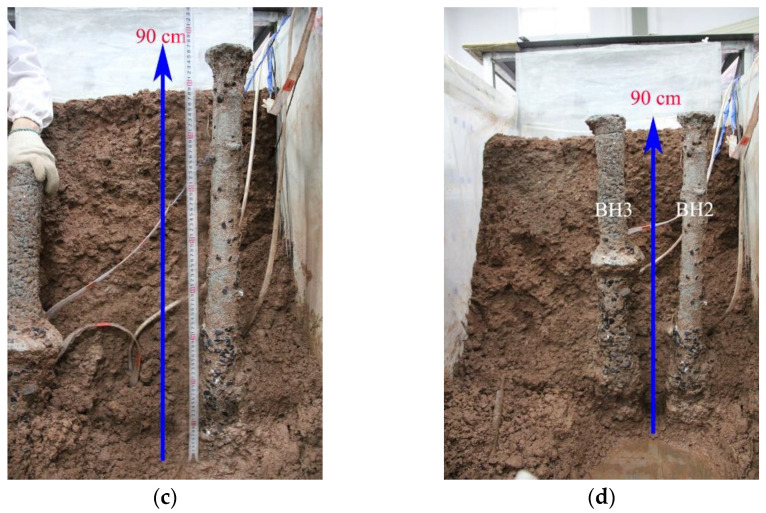
Cast in place pile formed after drilling with clean water and polymer fluid: (**a**) pile in BH2 formed by using water: (**b**) pile in BH3 formed by using water; (**c**) pile in BH2 formed by using polymer fluid; (**d**) pile in BH3 formed by using polymer fluid.

After the completion of drilling, boreholes formed with clean water and polymer fluid were covered with cement. After the cement was set, the bored piles were excavated, and the morphology of the piles was observed, as shown in Figure 10. As mentioned above, boreholes BH2 and BH3 had the same depth of 90 cm. Therefore, it was clear that from Figure 10a,b, when clean water was used to drill the boreholes, borehole collapse occurred in the lower parts of the BH2 and BH3 boreholes. The length of the pile formed in BH2 was 37 cm, so the length of the borehole collapse was 53 cm; the length of the pile formed in BH3 was 34 cm, so the length of the borehole collapse was 56 cm. However, as polymer fluid was used, the borehole did not collapse. The pile lengths of the BH2 and BH3 boreholes were the same as the borehole depth, both of which were 90 cm (Figure 10c,d).

In addition, from the appearance of the piles, when drilling with clean water, the piles were uneven, and the pile diameter changed considerably (Figure 10a,b). This phenomenon is due to the weak supportability of clean water as a borehole stabilizer, resulting in large radial displacement around the hole (Figure 9a) and the falling and collapse of the local block. In comparison, when polymer drilling fluid was used, the surfaces of the piles were basically flat, the change in the pile diameter was small (Figure 10c,d), and the radial displacements around the boreholes were small during drilling (Figure 9b). This was ascribed to the great supportability of the polymer fluid, which can maintain the stability of the borehole wall.

The mechanisms of polymer fluid stabilizing the borehole wall are as follows: (i) good lubrication performance. The polymer used in this paper was an acrylamide/sodium acrylate copolymer, which has the functions of flocculation and lubrication. It is conducive to controlling the solid phase of drilling fluid, and the friction resistance is relatively low. As a result, the borehole deformation caused by drilling is small; (ii) great supportability. The polymer fluid has a three-dimensional molecular network structure with intertwined and entangled chains (Figure 6). The polymer chain adsorbs on the surface of the borehole wall to form a dense adsorption membrane [21,33], which slows down the penetration rate of free water into the formation; (iii) the polymer chains form multi-point adsorption on the borehole wall and can cross the cracks, preventing the soil from peeling off and stabilizing the borehole wall [34].

It is worth noting that deionized water was used as the solvent instead of on-site construction water to ensure the reproducibility of the test results. However, it is acknowledged that the water at an actual job site will more or less contain dissolved salts, which could adversely affect the performance of the fluids. The results achieved in this paper can be regarded as the best for a given polymer tested under the stated conditions. In the future, on-site water should be adopted for research, which will help to further adjust and optimize the drilling fluid formula proposed in this article.

## 5. Conclusions

The present research work investigated the performance of PHPA polymer fluids to drill into a soil–limestone mixture. The fluid flow pattern, aging behavior, and the effect of polymer fluid mixed with silty clay were explored. A polymer fluid formula used in the mixture was proposed and verified. Based on the results presented in this paper, the following general conclusions were drawn:

1. An amount of 0.08% polymer fluid was a good alternative for the conventional 6% bentonite fluid, with 95 s and 89 s in Marsh funnel viscosity, respectively. Within the shear rate 5~1022 s^−1^, polymer fluids were reasonably well fitted to the power–law model, while bentonite fluids were suitable for the Bingham plastic model.
2. After a prolonged aging time of up to 30 days, the remaining active viscosity was at least 70%, which would promote confidence in utilizing polymer fluids as they will inevitably be placed for several days or even months at an actual job site.
3. Adding silty clay to the polymer fluids significantly reduced the apparent viscosity. As the dosage of silty clay increased from 3% to 10%, the rate of apparent viscosity reduction was from 50% to almost 76%, indicating the effective fluid management was to control silty clay on site during soil and rock mixture excavation.
4. A polymer drilling fluid formula: water + 0.08%PHPA + 0.1~0.5%Na_2_CO_3_ was proposed. Verified by drilling into a soi–limestone mixture, the formula has excellent lubrication performance and supportability, which causes small radial displacements around the boreholes and high drilling quality.

This present study broadens the research of PHPA polymer support fluids to drill into the material of soil and rock mixtures that are commonly faced in civil engineering, which would be helpful for consultants and contractors designing and constructing bored piles in soil and rock mixtures utilizing polymer fluids.

## Figures and Tables

**Table 1:** Physical properties of the soil-limestone mixture.

Dry Density (g/cm^3^)	Specific Gravity	Porosity (%)	*w* (%)	Grain Size Distribution (%)					
				20~40	10~20	5~10	2~5	0.005~2	<0.005
2.48	2.71	1.25	6	15	15	15	8	40	7

**Table 2:** Physical properties of the silty clay.

Dry Density (g/cm^3^)	Specific Gravity	*w* (%)	*w*_p_ (%)	*w*_L_ (%)	Grain Size Distribution (%)		
					<0.005	0.005~0.075	>0.075
1.67	2.72	14.2	15.1	29.1	15.0	38.2	46.8

## Data Availability

The data that support the findings of this study are available from the corresponding author, Peng Xia, upon reasonable request.
